# Hydrologic Response and Watershed Sensitivity to Climate Warming in California's Sierra Nevada

**DOI:** 10.1371/journal.pone.0009932

**Published:** 2010-04-01

**Authors:** Sarah E. Null, Joshua H. Viers, Jeffrey F. Mount

**Affiliations:** Center for Watershed Sciences, University of California Davis, Davis, California, United States of America; University of Bristol, United Kingdom

## Abstract

This study focuses on the differential hydrologic response of individual watersheds to climate warming within the Sierra Nevada mountain region of California. We describe climate warming models for 15 west-slope Sierra Nevada watersheds in California under unimpaired conditions using WEAP21, a weekly one-dimensional rainfall-runoff model. Incremental climate warming alternatives increase air temperature uniformly by 2°, 4°, and 6°C, but leave other climatic variables unchanged from observed values. Results are analyzed for changes in mean annual flow, peak runoff timing, and duration of low flow conditions to highlight which watersheds are most resilient to climate warming within a region, and how individual watersheds may be affected by changes to runoff quantity and timing. Results are compared with current water resources development and ecosystem services in each watershed to gain insight into how regional climate warming may affect water supply, hydropower generation, and montane ecosystems. Overall, watersheds in the northern Sierra Nevada are most vulnerable to decreased mean annual flow, southern-central watersheds are most susceptible to runoff timing changes, and the central portion of the range is most affected by longer periods with low flow conditions. Modeling results suggest the American and Mokelumne Rivers are most vulnerable to all three metrics, and the Kern River is the most resilient, in part from the high elevations of the watershed. Our research seeks to bridge information gaps between climate change modeling and regional management planning, helping to incorporate climate change into the development of regional adaptation strategies for Sierra Nevada watersheds.

## Introduction

General circulation models (GCMs) predict an increase in air temperature across California's Sierra Nevada mountain range, although predictions vary whether the region can expect more or less precipitation [1], [2]. Most studies agree that decreases in mean annual flow, reduced snowpack, and more rapid snowmelt runoff are expected [3], [4], [5], [6]. However, it is not well understood whether individual watersheds within a single region will respond differently to climate warming, how characteristics of the individual watersheds may temper future impacts, and how differential impacts relate to existing demands such as water storage capacity, hydropower generation, and ecosystem services.

In this paper, we analyze model results from 15 neighboring watersheds to examine differential watershed response within a larger region. We use results from a climate-forced rainfall-runoff model to explicitly simulate intra-basin hydrologic dynamics and understand localized sensitivity to climate warming. Insights presented here are intended to help guide local adaptation strategies by highlighting regional and basin-specific trends in the quantity and timing of water resources under regional climate warming, and to illustrate which basins are the most intrinsically vulnerable to climate warming.

Due to uncertainty regarding future precipitation change [1], we assume a historic hydrology and focus singularly on hydrologic response to climate warming. We analyze climate warming effects at the watershed scale for 15 west-slope watersheds of the Sierra Nevada mountain range. Model domain extends from the crest of the Sierra Nevada to the floor of California's Central Valley. Climate sensitivity analyses include basecase unimpaired conditions and uniform air temperature increases of 2°C, 4°C, and 6°C to bracket the range of likely outcomes for Sierra Nevada watersheds with climate warming. Other climate variables are unchanged from historic values. The modeled period, water years 1981–2001, covers a wide range of climatic variability including the wettest year on record (1983), the flood year of record (1997), and a prolonged drought (1988–1992). Predicting the frequency of extreme events due to climate warming is outside the scope of this study. Results are interpreted by focusing on potential impacts of changed water yield to water storage, runoff timing to hydropower generation, and extension of low flow duration to montane ecosystems, such as high elevation meadows, riparian areas, and aquatic habitats.

The Sierra Nevada mountain range is a water source for many of California's 38 million residents. The region has been extensively developed for water resources with reservoirs and conveyance facilities to enhance water supplies, hydropower, and flood control for downstream communities. Environmental minimum instream flows maintain habitat for aquatic and riparian ecosystems, and rivers and reservoirs are also used extensively for recreational purposes.

Climate warming will alter Sierra Nevada water resources in a number of ways, but direct impacts to water supply, hydropower generation, and montane ecosystems are likely to be profound. Most climate modeling for hydrologic impacts in California has focused on global or regional trends with fairly coarse resolution [1], [2], [6], [7], [8], [9] or on individual watersheds using finer resolution [4], [5], [10], [11]. Regional response across watersheds has not been synthesized in a systematic way, making planning difficult at the watershed or local level [7], [12]. For this reason, climate warming is often excluded from local and regional planning efforts, the scales most appropriate for anticipating impacts and developing adaptation strategies.

This paper begins with a description of the 15 modeled west-slope watersheds of the Sierra Nevada with respect to current water supply, hydropower generation, and montane ecosystems. We present input data, assumptions, and governing equations for the Water Evaluation and Planning System (WEAP21) Sierra Nevada unimpaired hydrologic model, and discuss results of warming scenarios relative to basecase conditions. For each watershed, we present changes in mean annual flow (MAF), centroid timing (CT), and low flow duration (LFD) to highlight relative differential responses across basins, and in relation to water resource development (i.e., water delivery, hydropower, and mountain meadows). Our findings suggest anticipated hydrologic changes from climate warming to the western Sierra Nevada are heterogeneous and that relative risk to water resources is non-uniform. The American, Yuba, and Feather watersheds have large reductions in water yield with climate warming and are also important for water supply. The Stanislaus, Kings, and San Joaquin basins have major shifts in runoff timing combined with the most hydropower generating capacity of all the basins. The Mokelumne, Tuolumne, and Stanislaus have substantial increases in the length of late summer low flow conditions and also have the most mountain meadows, which are vulnerable to such changes.

### Study Area

**Figure 1:**
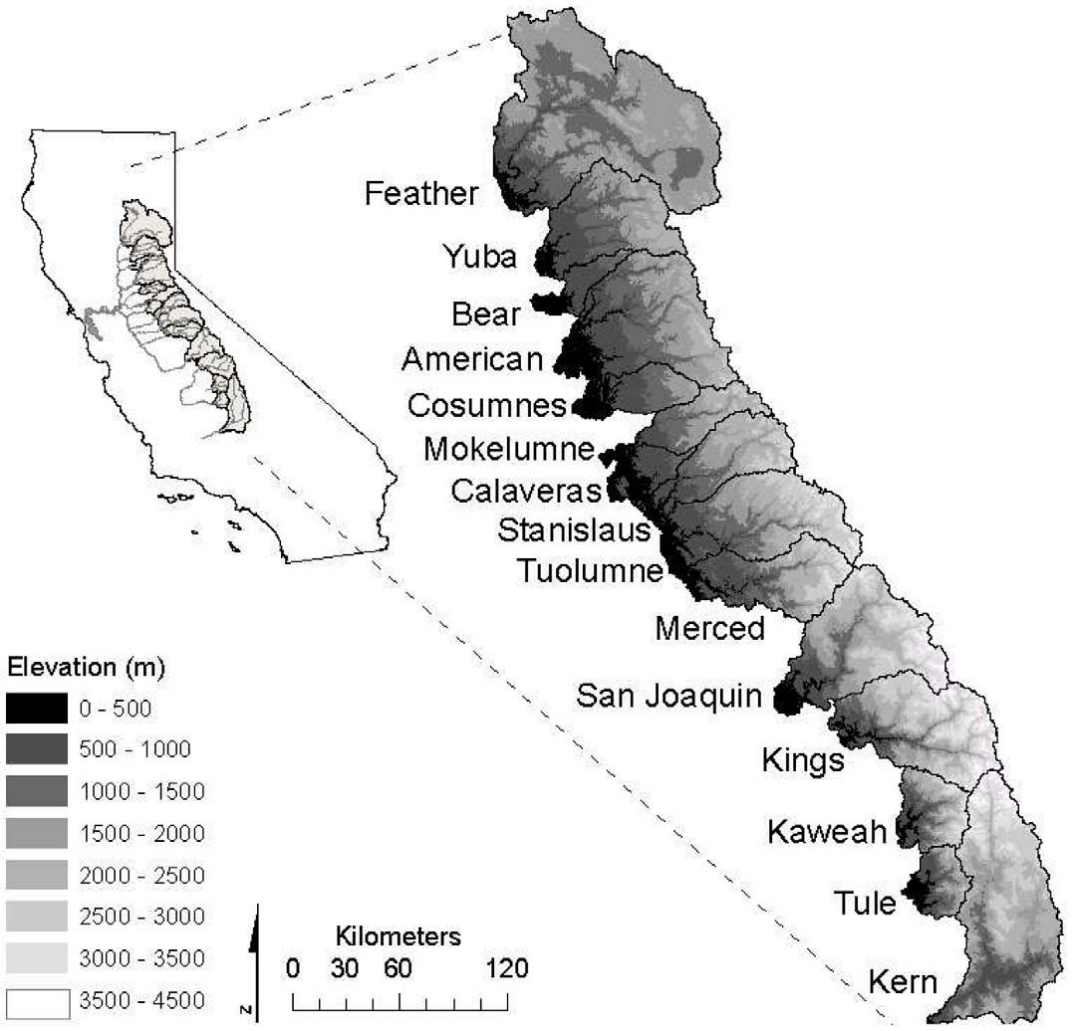
Project watersheds and topography.

California's Sierra Nevada mountain range is oriented generally north-south, separating California's Central Valley from the Basin and Range province to the east. West slope Sierra Nevada rivers flow generally westward to their confluence with the Sacramento or San Joaquin Rivers, which then merge and flow through the San Francisco Bay Delta to the Pacific Ocean (Figure 1).

#### Climate and Hydrology

The Sierra Nevada is characterized by a Mediterranean-montane climate with a distinct cool, wet season from November to April and a warm, dry season from May to October. During the dry season, precipitation is infrequent except for high elevation thunderstorms (>3,000m). During the wet season, precipitation falls as both snow and rainfall, and the snowline is approximately 1,000 m. Precipitation averages approximately 108 cm/yr for the region, although it is highly variable due to elevation, latitude, and local weather patterns (Table 1). The Feather, Yuba, and American watersheds have the highest precipitation rates, where maximum precipitation exceeds 200 cm/yr [13].

**Table 1:** Physical watershed characteristics (north to south).

Watershed	Abbreviation	Area (km^2^)	Mean Precip. (cm/yr)	Precip. Range (Min–Max) (cm/yr)	Elevation Range (m)	Northing Centroid (km)	Max Strahler Stream Order
Feather	FEA	9,412	121.5	36.6–301.4	275–2,853	4,425	7
Yuba	YUB	3,114	167.5	83.2–223.6	76–2,772	4,370	6
Bear	BAR	730	122.1	63.2–187.0	90–1,772	4,334	5
American	AMR	4,822	135.8	63.0–203.6	39–3,163	4,313	7
Cosumnes	COS	1,385	107.3	58.9–143.4	55–2,359	4,275	6
Mokelumne	MOK	1,498	123.3	57.8–164.3	72–3,162	4,261	6
Calaveras	CAL	937	86.5	55.3–142.8	212–1,851	4,231	5
Stanislaus	STN	2,341	115.9	64.8–168.1	211–3,520	4,238	6
Tuolumne	TUO	3,971	110.1	43.5–172.8	245–3,989	4,206	6
Merced	MER	2,685	104.5	50.1–159.3	245–3,990	4,174	6
San Joaquin	SJN	4,315	101.4	35.5–159.1	97–4,224	4,139	6
Kings	KNG	3,998	96.4	50.1–154.5	177–4,349	4,094	6
Kaweah	KAW	1,451	94.0	36.8–151.1	154–3,846	4,047	6
Tule	TUL	1,015	76.4	28.6–119.2	174–3,119	4,008	6
Kern	KRN	5,983	56.0	24.4–147.3	171–4,418	3,992	7

#### Geography

Modeled watersheds from the Feather River watershed in the north to the Kern River watershed in the south encompass a total area of 47,657 km^2^ and span approximately 628 km. Historic mean annual unimpaired runoff from 1981–2001 for the 15 modeled watersheds was approximately 26,234 mcm (million cubic meters) [14]. Basins vary greatly by size: the Feather River watershed is the largest, and the Bear River watershed the smallest (Table 1). Total linear stream length mirrors watershed size, as the Feather River watershed and the Bear River watershed also have the longest and shortest total kilometers of streams, respectively.

The southern portion of the Sierra Nevada is generally higher, with elevations greater than 4,000 m at the crest, while the northern watersheds are generally less than 3,000 m at peak elevations. The 4,418 m peak of Mt. Whitney in the Kern River watershed is the highest point of all study watersheds. Most Sierra Nevada watersheds are steep at their headwaters, with slope generally decreasing toward the alluvial Central Valley. The snowpack of the Sierra Nevada acts as a natural reservoir, storing water during winter and melting throughout spring. Historically, approximately 18,500 mcm of California's water was from snowmelt; although that volume is predicted to decrease with climate warming in coming decades [15]. The geography of the state allows water suppliers to provide clean, gravity-fed water from the Sierra Nevada to large urban centers, generating hydropower in the process. Major Sierra Nevada water projects in this region include the federally funded Central Valley Project, California's State Water Project, San Francisco's Hetch Hetchy System, and San Francisco East Bay Area's Mokelumne Aqueduct.

#### Water Resource Development

In general, watersheds of the west-slope Sierra Nevada are extensively developed for water resources. For the 15 watersheds included in this study, total water storage is approximately 24,590 mcm for all dams greater than 1.2 mcm (1 taf) [16], and total online hydropower capacity is approximately 8,751 MW [17] (Table 2). Many of the larger reservoirs and water projects located in the Sierra Nevada are operated for multiple uses, such as water supply, hydropower, flood control, environmental mitigation, and recreation. Larger reservoirs at the lower elevations are operated primarily for water supply and flood control, while smaller reservoirs at upper elevations are operated mainly for hydropower generation. Climate warming is expected to affect high elevation dams operated for hydropower differently than low elevation dams operated primarily for water supplies [18].

**Table 2:** Water resource benefits by watershed (north to south).

Watershed	Total Online Capacity (MW)	Hydropower Facilities	FERC Relicenses (next 40 yrs)	Total Water Storage Capacity (mcm)	Number of Dams (>1taf)	Wild and Scenic Rivers (km)	Human Population (2000 census)
Feather	1,635	23	7	6,668	25	125	34,634
Yuba	424	12	4	1,764	22	–	32,699
Bear	257	15	1	224	5	–	54,978
American	1,221	19	5	2,216	24	99	95,883
Cosumnes	0	0	0	51	1	–	24,201
Mokelumne	374	7	2	1,050	13	–	7,115
Calaveras	2	1	1	394	2	–	11,563
Stanislaus	1,010	12	7	3,505	12	–	15,847
Tuolumne	558	6	1	3,352	9	134	44,663
Merced	108	3	2	1,285	2	197	6,238
San Joaquin	1,278	17	5	1,566	12	–	9,907
Kings	1,715	6	4	1,536	6	130	2,073
Kaweah	26	4	2	176	1	–	2,443
Tule	10	3	3	102	1	–	4,709
Kern	133	6	5	701	1	243	14,661

## Methods

Modeling was completed using the Stockholm Environment Institute's Water Evaluation and Planning System (WEAP21), a spatially explicit rainfall-runoff model. WEAP21 operates on a weekly timescale, and simulations were completed for 1981–2001 historical hydrology [14]. WEAP21 models the terrestrial water cycle to represent physical hydrology using a one-dimensional, two-storage soil compartment water balance.

Watersheds, subwatersheds, and elevation bands were delineated using USGS digital elevation models (DEMs). Next, subwatersheds and 250 m elevation bands from the crest of the Sierra Nevada to the floor of California's Central Valley were intersected to create smaller land units, termed catchments here. Elevation bands were used to provide resolution in the snow accumulation range of the Sierra Nevada. Land cover vegetation affects evapotranspiration (ET) and soil depth affects soil moisture capacity, so within catchments vegetation was classified using GIS data from the National Land Cover Dataset and soil depth was classified using SSURGO and STATSGO data (Table 3). Finally, the areas within each catchment of all land cover – soil combinations were determined. Snow accumulation, snowmelt, runoff, soil moisture storage, evapotranspiration, interflow, deep percolation, and baseflow were then calculated for each area using the equations below. This application of WEAP21 uses 1,268 catchments with an average area of 37.6 km^2^ [14], [19].

**Table 3:** WEAP21 input data and sources.

*Input Data*	*Source*
Meteorology	DAYMET
Vegetative Land Cover	National Land Cover Dataset (NLCD)
Soil Depth	Natural Resource Conservation Service (NRCS) SSURGO or STATGO
Topography	USGS 10 m DEM
Calibration – Snow Water Equivalent	CDWR
Calibration – Estimates of Unimpaired Flows	CDWR

**Figure 2:**
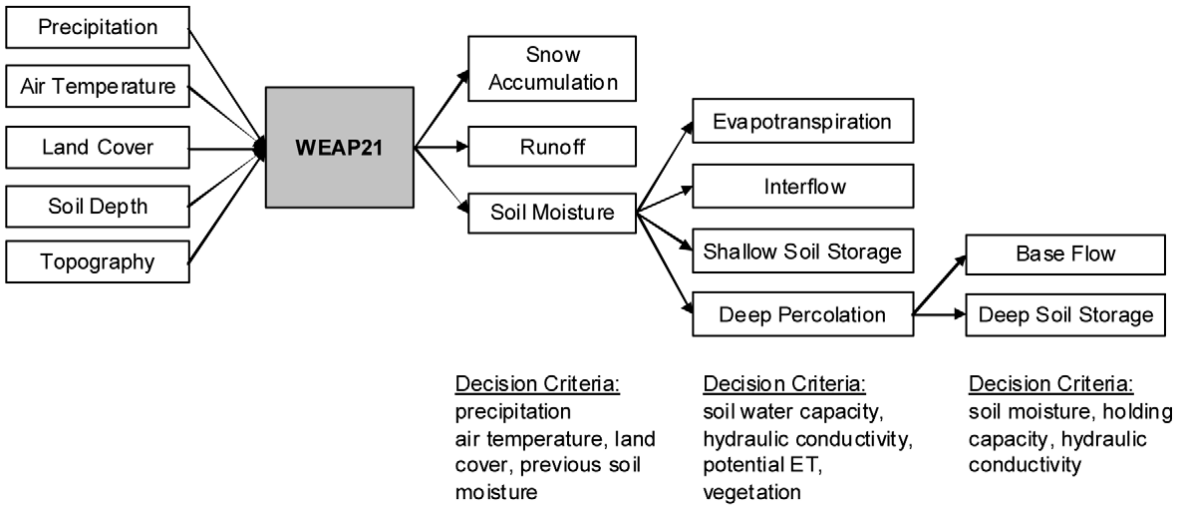
WEAP21 rainfall-runoff model flow chart.

Precipitation is partitioned as snow, runoff, or infiltration depending on air temperature, land cover, soil depth, and previous soil moisture conditions. Shallow soil moisture is further partitioned into evapotranspiration, interflow, deep percolation, or storage based on soil moisture capacity, hydraulic conductivity, potential ET, and land cover specific ET coefficients. Deep percolation can enter a second deep soil compartment as either base flow or deep soil moisture (Figure 2). The mass balance for soil moisture storage in the upper soil layer (S_w_) for each land cover (j) is
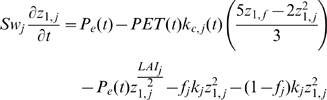
(1)where z_1,j_ is relative soil water storage (varies between 0–1), t is time (on a weekly time step), P_e_ is effective precipitation (mm), PET is evaporation from the area using the Penman-Monteith reference crop potential evapotranspiration (mm/day), k_c_ is the plant coefficient, LAI is a leaf and stem area index value, f is a calibration parameter for soil, land cover, and topography that partitions water horizontally (f_j_) or vertically (1−f_j_), and k is a parameter used to estimate upper soil storage conductivity.

The first term in equation 1 is effective precipitation as a function of total precipitation, snow accumulation, and snowmelt, and is explained further below. The second term is evapotranspiration, the third term is surface runoff, the fourth term is interflow, and the fifth term represents deep percolation. Baseflow, Bf(t), is simply calculated as
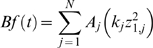
(2)where A is the contributing area of each land cover class (m^2^).

Effective precipitation, P_e_, is calculated with an imbedded temperature-index snowmelt model
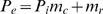
(3)where P_i_ is observed total precipitation, m_c_ is a snowmelt coefficient dependent on observed air temperature, melting, and freezing temperatures, and m_r_ is melt rate which is a function of snow accumulation, the snowmelt coefficient, and the available melt energy. See Yates et al. [19] and Young et al. [14] for a full description of the temperature-index snowmelt model and additional model detail.

In addition to the 10 m DEMs, vegetation land cover, and soil depth input data discussed above, climate data (air temperature, precipitation, and vapor pressure deficits) for the 1981–2001 period were used to generate modeled hydrology (Table 3). Interpolated weather data from DAYMET was used to represent temperature and precipitation variability caused from orographic effects and because adequate measured data were unavailable (stations were sparse in the Sierra Nevada). Daily DAYMET data has a spatial resolution of 1 km^2^, and climate data was obtained for a single location within each catchment (near the centroid of the catchment on the mid-elevation contour) [14]. Climate conditions are assumed to be uniform within each catchment, the smallest spatial unit of analysis, but vary between catchments.

Unimpaired historic hydrology and uniform air temperature increases of 2°C, 4°C, and 6°C (results are labeled as basecase, T2, T4, and T6, respectively) were modeled as a sensitivity analysis of discharge characteristics with respect to temperature [14]. There is general agreement among GCMs that California's climate is warming, although the extent of warming is not consistent among models, with some ensembles resulting in drier conditions and some in wetter [1], [2]. Although the volume of precipitation is unchanged from historic conditions, increasing air temperature can change the form of precipitation (typically from snowfall to rainfall), which is discussed with model results. Climate warming also alters rate of evapotranspiration, soil storage, and snowmelt timing which changes discharge characteristics in study watersheds.

Our three warming scenarios represent progressively severe warming (or alternatively modest warming over a progressively longer outlook). For perspective, average annual 2°C warming roughly represents climate warming projections from HadCM3, a medium sensitivity U.K. Met Office Hadley Centre Climate Model, using the A1fi (higher emissions) scenario for 2020–2049 (and also approximately represents projections from PCM, a low sensitivity National Center for Atmospheric Research/Department of Energy Parallel Climate Model, using the B1 (lower emissions) scenario for 2070–2099). Average annual 4°C warming approximately represents projections from 2070–2099 PCM climate change using the B1 scenario, and average annual 6°C represents projections from 2070–2099 HadCM3 climate change using the A1fi scenario [2]. In this study, we assumed uniform increases in air temperature; however, previous modeling efforts have shown that larger increases in air temperature are expected during summer with smaller increases during winter [2]. Regardless, sensitivity analysis using uniform climate input data is common for localized climate modeling to bookend the range of hydrologic responses from climate change [5], [11], [20], [21].

### Model Testing

Each watershed was calibrated with monthly unimpaired streamflow estimates and snow water equivalent measurements from the California Department of Water Resources (CDWR) [14]. Measured data at finer temporal resolution were unavailable for several watersheds. Overall, the models mirrored the major features of flow hydrographs at watersheds outlets (Table 4). RMSE at watershed outlets weights high flows more than low flows, and the worst fit occurred in the San Joaquin, Mokelumne, and Tule watersheds. RMSE_log_ weights low flows more than high flows and the worst fit occurred in the Cosumnes, Mokelumne, American, Stanislaus, and Tule watersheds [14]. Additional verification was completed at the subwatershed scale using measured flow from USGS gages at unregulated streams to assess intra-basin performance [14], but is not discussed here as we focus only on flow magnitude and timing changes at the terminal outlet of each basin (mean outlet elevation is 153 m).

**Table 4:** Goodness of fit statistics for predicted monthly full natural flows at terminal outlets (WY 1982–2000, n = 228) (from [14]).

Watershed^t^	Bias (%)*	RMSE (%)**	RMSE_log_ (%)
Feather	0	53	1
Yuba	0	47	3
American	0	39	14
Cosumnes	−3	38	29
Mokelumne	0	60	18
Stanislaus	2	54	14
Tuolumne	−5	55	4
Merced	1	50	10
San Joaquin	−4	65	3
Kings	−6	49	3
Kaweah	0	42	2
Tule	−2	58	14
Kern	−2	54	2

t Monthly estimated streamflows were unavailable for the Bear and Calaveras watersheds (those basins calibrated only with SWE data).

*Bias = 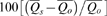.

**RMSE (root mean square error) = 
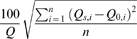
, where Q_s,i_ and Q_o,i_ are simulated and observed flow rates for each time step, (i).

### Limitations

Validating hydrologic models used for climate change predictions or sensitivity analyses are not truly possible until measurable climate changes actually occur [22]. The model used here has been calibrated for each watershed modeled, and has also been validated using measured flow collected by USGS from unimpaired subwatersheds, demonstrating intra-basin model performance. A differential split-sample calibration was not completed (where we calibrated using the wettest years in our domain, and validated using the driest years) [23]. However, precipitation during the study period is highly variable with the 4^th^, 8^th^, and 10^th^ driest years on record, and the 1^st^, 2^nd^, and 4^th^ wettest years on record, according to the CDWR Sacramento Valley water year classification index. The climate variability of the calibration period helps to ensure goodness of fit to use this model for climate warming sensitivity analysis. Since the rainfall-runoff model is implemented as a sensitivity analysis to incremental air temperature increases, the results presented here are not predictions, but rather assessments of watershed resiliency to increased air temperature, and provide bookends for the range of possible outcomes for water resource management in Sierra Nevada watersheds.

## Results

### Mean Annual Flow (MAF)

The total reduction in mean annual flow (MAF) from the Sierra Nevada region is important for future water supply and hydropower planning decisions, as well as protection of aquatic ecosystems at the regional and watershed levels. We predicted an overall trend toward reduced MAF, mostly from higher evapotranspiration with climate warming. The 1997 water year was a deviation from this trend due in large part to a large rain on snow storm event, when rainfall occurred quickly without time to recharge soil moisture.

Overall, total MAF from all watersheds was reduced with climate warming (Table 5). The quantity of water reduced from each 2°C air temperature increase was roughly similar, suggesting there was no threshold which drastically reduces annual runoff when the climate warms as much as 6°C. Each 2°C increase in air temperature led to a total reduction of nearly 700 mcm of the mean annual flow for the Sierra Nevada region (the sum of all study watersheds). Thus, 2°C, 4°C, and 6°C air temperature increases resulted in decreases of approximately 633 mcm (the size of Millerton Lake in the San Joaquin River Basin), 1,324 mcm (the size of New Exchequer's Lake McClure in the Merced River Basin), and 2,074 mcm (half the size of Lake Oroville in the Feather River Basin), respectively.

**Table 5:** MAF by climate alternative and watershed (T indicates modeled temperature, with increases of 2, 4, and 6°C).

Watershed	Annual Average Flow (mcm)				Change from Basecase (%)		
	Basecase	T2	T4	T6	T2	T4	T6
Feather	5776	5649	5470	5264	2.2	5.3	8.8
Yuba	3020	2960	2891	2806	2.0	4.3	7.1
Bear	492	475	459	445	3.6	6.7	9.6
American	3556	3448	3332	3218	3.1	6.3	9.5
Cosumnes	603	571	543	518	5.2	10.0	14.0
Mokelumne	979	946	918	887	3.4	6.2	9.4
Calaveras	330	319	310	301	3.3	6.3	8.9
Stanislaus	1561	1523	1482	1435	2.4	5.1	8.1
Tuolumne	2445	2401	2354	2304	1.8	3.7	5.8
Merced	1348	1308	1272	1237	3.0	5.6	8.2
San Joaquin	2294	2265	2235	2201	1.3	2.6	4.1
Kings	2117	2094	2070	2041	1.1	2.2	3.6
Kaweah	586	564	542	519	3.8	7.6	11.5
Tule	199	190	180	171	4.6	9.5	14.3
Kern	926	887	850	813	4.2	8.2	12.2

Our results are broadly consistent with other climate forecasts. Brekke et al. [9] report that Sacramento and San Joaquin valley floor reservoir inflows will decrease by 5% by 2025, and 14% by 2065 using a PCM warm and dry climate alternative. Our results indicate an average 3%, 6%, and 9% annual flow reduction for study watersheds with 2°C, 4°C, and 6°C warming, respectively. These results are also consistent with climate change impacts from Lettenmaier and Gan [10] and Miller et al. [5].

The year with the largest reduction in MAF for the Sierra Nevada region was 1998 for 2°C warming, with 1,345 mcm less flow. For 4°C and 6°C warming, 1995 had the greatest total flow reduction, with 2,962 mcm and 4,540 mcm less flow, respectively. Both 1995 and 1998 were classified as wet years using the Sacramento and San Joaquin Valley Water Year Indices, and 1998 was an El Niño year [24]. Climate warming increased MAF slightly for 1997, another wet year with substantial flooding in some watersheds due to rain on snow storm events. In 1997, the 2°C, 4°C, and 6°C warming alternatives increased total flow by 385 mcm, 539 mcm, 371 mcm, respectively. WEAP21 estimated a gain in flow because intense, wet storms did not provide time for water to infiltrate soil or be stored as snowpack. Thus, results suggest climate warming will cause a shift from snowfall to rainfall over more area and from more storms causing runoff to be flashier and sometimes with higher flow magnitudes, but less water will be stored within watersheds.

**Figure 3:**
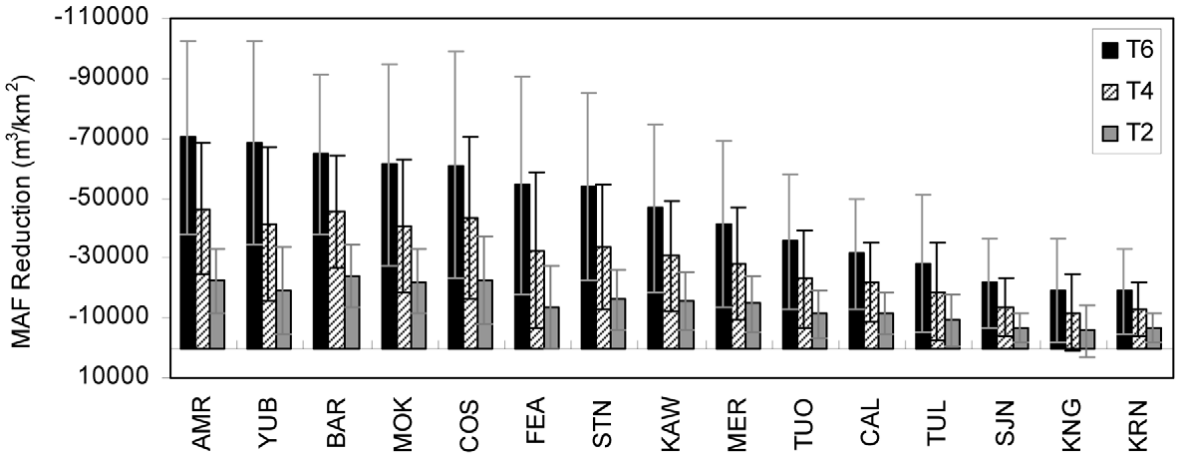
MAF reduction by watershed and climate alternative with 21 year standard deviation bars.

**Figure 4:**
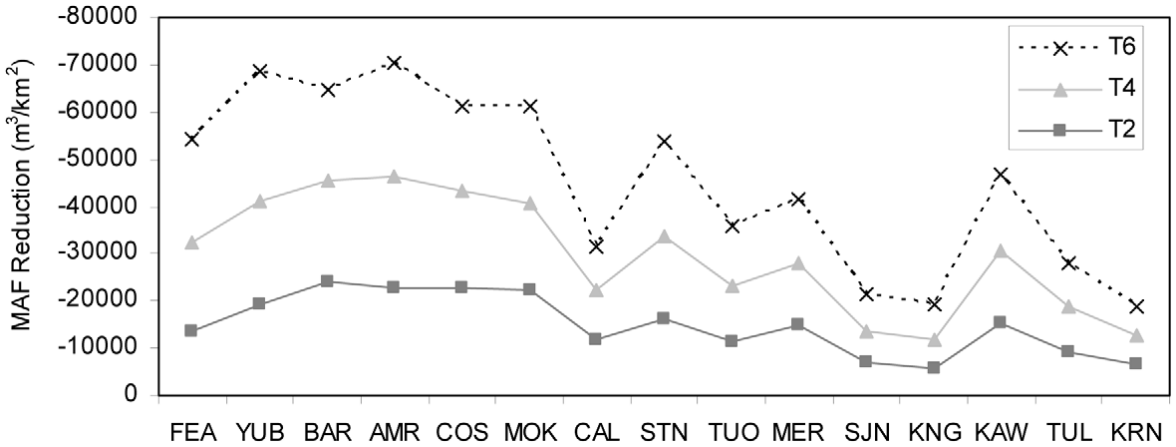
MAF reduction from basecase by watershed and climate alternative (north to south).

There was considerable variability in MAF between basins, which is largely a function of watershed area (Table 5). When change in MAF was normalized by area, the Bear River had the largest MAF change for 2°C climate warming, with a 24,107 m^3^/km^2^ reduction; and the American River had the most change for 4°C, and 6°C warming, with 46,458 m^3^/km^2^ and 70,167 m^3^/km^2^ less flow, respectively (Figure 3). Overall, watersheds in the northern Sierra Nevada had greater reductions in MAF from climate warming (Figure 4). In WEAP, change in MAF was driven by evapotranspiration. The northern Sierra Nevada had more trees and less shrub land cover than the southern Sierra Nevada. Additionally, watersheds in the southern portion of the range had more barren land at upper elevations. We assumed land cover did not change with climate warming.

**Figure 5:**
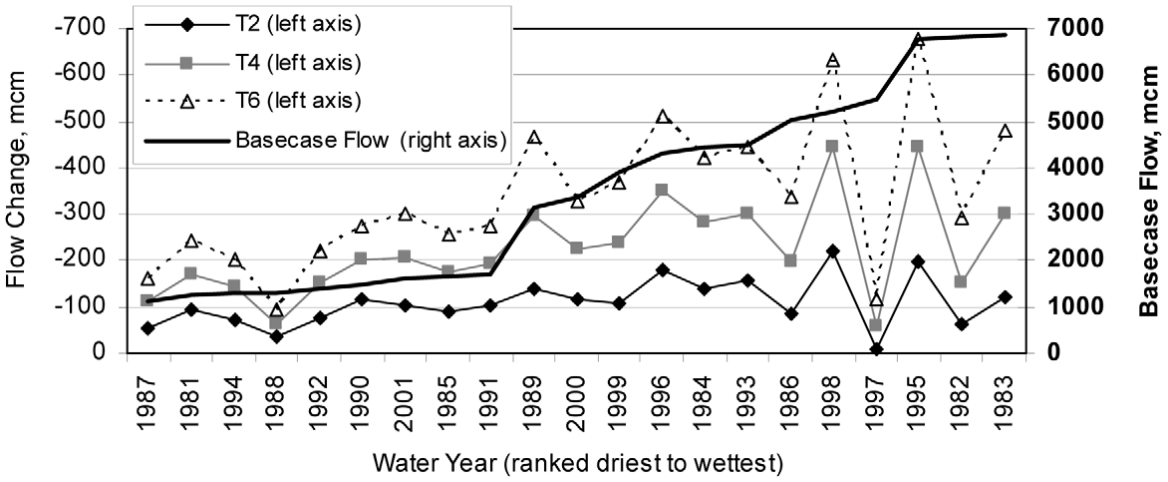
American River average flow change by climate alternative.

Yearly estimated change in flow from climate warming is presented for the American River as an example of hydrologic variability within watersheds (Figure 5). These results show that hydrologic variability between years increased with climate warming, even with no change in precipitation input data.

### Runoff Centroid Timing (CT)

Runoff centroid timing (CT) is the date at which half of the annual runoff at the outlet of each watershed has passed. It was calculated as
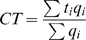
(4)where t_i_ is time in weeks from the beginning of the water year, and q_i_ is streamflow for week i [7]. CT is a date given as a Julian week for a water year, so 1 is the first week of October, and 52 is the last week in September.

**Figure 6:**
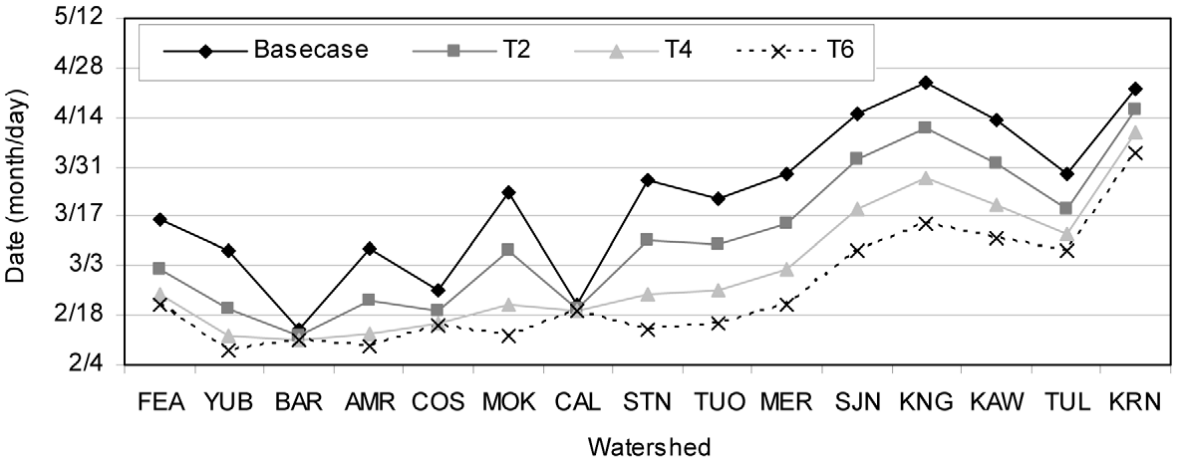
Average annual CT by watershed and climate alternative (north to south).

With climate warming, average annual CT occurred earlier in the year, and there was less variability in timing between watersheds (Figure 6). Southern-central Sierra Nevada watersheds (Stanislaus to Kaweah) had equal length timing shifts with each 2°C air temperature increase. This was not the case in the watersheds of the northern Sierra Nevada that reached the Sierra crest (excluding the Bear, Cosumnes, and Calaveras watersheds) where timing shifts were shorter between T4 and T6 than they were between basecase and T2. CT was primarily driven by snowmelt. These results illustrate that there was little remaining snowpack in northern watersheds that reach the Sierra crest when air temperature was increased by 4°C, thus there was little change between T4 and T6. The southern Sierra Nevada is higher and retains more of its snowpack, so CT continues shifting to earlier dates between T4 and T6.

Low elevation watersheds that do not reach the crest of the Sierra Nevada, such as the Bear, Cosumnes, and Calaveras Rivers, experienced little change in timing. These watersheds have less snowfall (and thus less snowmelt) and less area to drain than other basins, both of which are stabilizing factors for CT. The Kern River also had little change in CT with climate warming. It has the highest crest elevation of all watersheds and although results showed reduced snowfall from climate warming, snowmelt continued later in spring due to colder temperature at upper elevations. Thus, very high elevation watersheds that maintain cooler air temperatures and low watersheds that already have less snowpack are more resilient to CT than northern Sierra Nevada watersheds that reach the crest of the range. Climate warming will most likely shift precipitation from snowfall to rainfall with earlier snowmelt, resulting in much earlier runoff than historic conditions.

**Figure 7:**
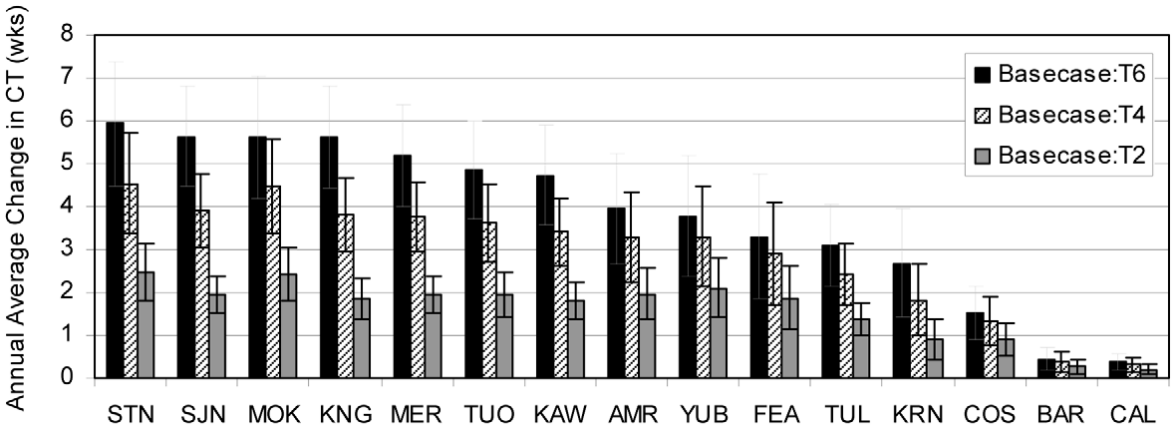
Average annual CT change by watershed and climate alternative with 21 year standard deviation bars.

The Stanislaus River had the greatest change in CT from basecase conditions, although results indicate the San Joaquin, Mokelumne, Kings, and Merced Rivers also had CT shifts approximately five to six weeks earlier in the year with a 6°C rise in air temperature (Figure 7). For every 2°C rise in air temperature, average CT occurred nearly 2 weeks earlier in those basins. For example, at the outlet of the Stanislaus River under basecase conditions average CT occurred approximately March 27, but was estimated to occur March 10, February 24, and February 14 with 2°C, 4°C, and 6°C warming, respectively (Figure 6). (We discuss results here as days rather than fractions of weeks to make results more easily understandable. However, our model operates on a weekly timestep, and timing changes should be interpreted relative to other watersheds, rather than precise calendar dates.) Average timing for the Tuolumne River was approximately the same as the Stanislaus River. Centroid timing occurred later in the San Joaquin and Kings watersheds than in the Tuolumne and Stanislaus watersheds under all scenarios (Figure 6), most likely due to the high elevations in the San Joaquin and King Rivers, resulting in comparatively late runoff. In the Kings watershed, average CT occurred approximately April 24 under basecase conditions, and shifted to April 11, March 28, and March 15 with 2°C, 4°C, and 6°C warming. The Bear and Calaveras watersheds had the smallest runoff timing shift observed, with average CT approximately one day earlier for each 2°C rise in air temperature.

### Low Flow Duration (LFD)

Low flow duration (LFD) is the number of weeks with low flow conditions. Low flow weeks were counted if weekly discharge normalized by total discharge for a water year was less than 1% of the total discharge from that water year.
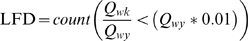
(5)where Q_wk_ is discharge for a week, and Q_wy_ is total discharge for a water year (sensu. [25]). We further constrained LFD to be at least three consecutive weeks. Low flow conditions lasting three weeks or longer primarily occurred in summer to early fall, the time typically associated with low flows. This method removed isolated weeks when flows decreased, but soil moisture remained high, providing plenty of water for evapotranspiration and groundwater. Overall, this approach worked well, although it over-predicted LFD in years with large floods, such as 1997, because summer baseflow remained approximately the same as years with more average total discharge.

**Figure 8:**
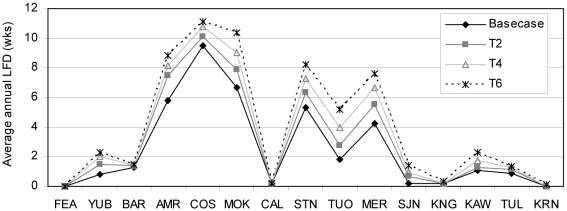
Average annual LFD by watershed and climate alternative (north to south).

Persistent low flow conditions are detrimental to water supply and montane ecosystems, and it is during this period that water demands are highest relative to supply. Climate warming lengthened this critical time for many watersheds, particularly those in the central Sierra Nevada. The Cosumnes River had the most weeks with low flow conditions at its terminal outlet under all climate alternatives (Figure 8). For that watershed, average LFD was 9.5 weeks with basecase conditions 10.1 weeks with 2°C warming, 10.8 weeks with 4°C warming, and 11.2 weeks with 6°C warming. The Feather River did not experience low flow conditions with any climate alternatives, and is the basin with the most groundwater, thus it is most resilient to low flow conditions.

**Figure 9:**
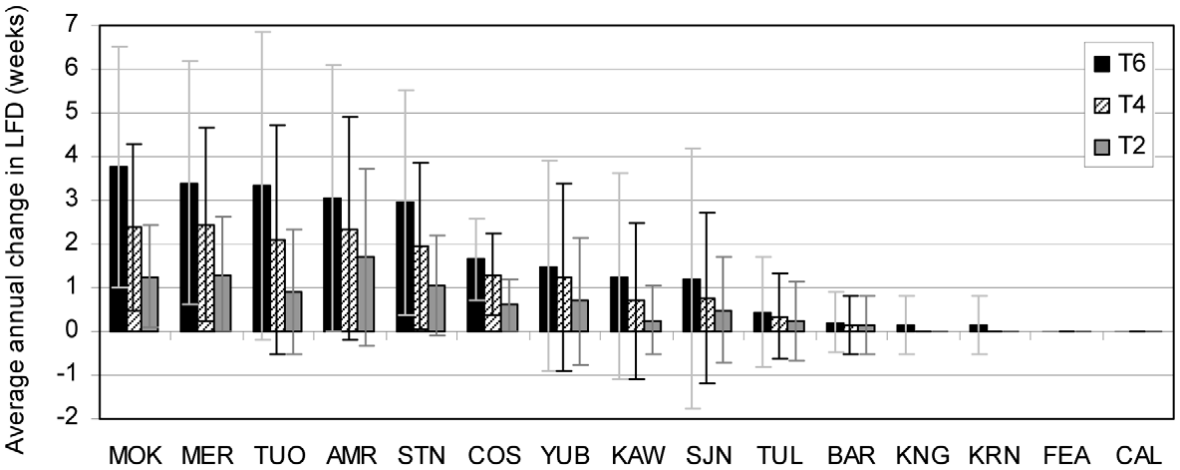
Average annual LFD change by watershed and climate alternative with 21 year standard deviation bars.

The Mokelumne, Merced, Tuolumne, American, and Stanislaus watersheds had the most change in average LFD from basecase conditions as a result of climate warming (Figure 9). LFD is inversely related to deep soil moisture storage, so LFD is short or absent when deep soil moisture storage is near capacity. In the above basins, the water in the deep soil layer was consistently less than 10% of its holding capacity during mid summer to early fall (July through October). Those watersheds experienced approximately one more week of LFD for each 2°C increase in climate warming. The Tule and Kern watersheds also had low soil moisture (<10% of holding capacity), but results indicated low flow conditions did not occur using the method for calculating LFD discussed above. In the Tule and Kern watersheds, low flow conditions exist when Q_wk_/Q_wy_<2%. Further research is needed to better define LFD for all watersheds and all year types.

The Mokelumne River had the greatest increase in LFD weeks (from basecase conditions to 6°C climate warming) in 1982 and 1983, both wet years. There was no increase in LFD weeks with climate warming in 1981 and 1994, a dry and critically dry year. This suggests that as precipitation shifts from snowfall to rainfall, summer and autumn flows during wet years will be relatively drier as a result of flashier storms that do not replenish soil moisture from snowmelt.

### Regional Climate Warming Changes

**Figure 10:**
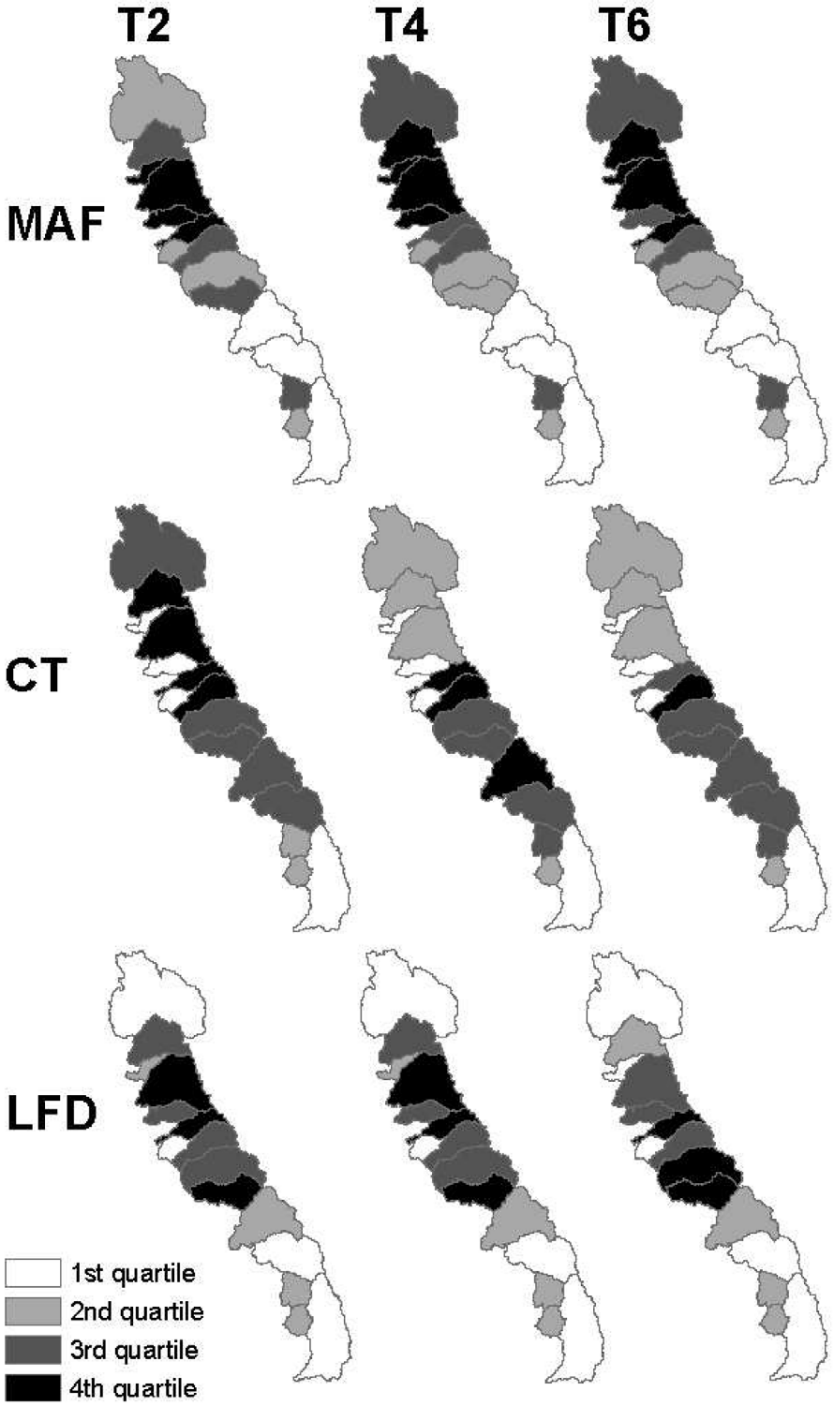
Change in MAF (m^3^/km^2^), CT (wks), and LFD (wks) from basecase conditions by watershed and climate alternative.

Results suggest that climate warming affects watersheds differently for MAF, CT, and LFD, which could have repercussions for water supply, hydropower, and ecosystem services. Overall, the northern Sierra Nevada had the most change in MAF, the high watersheds of the southern-central Sierra Nevada had the most change in CT, and the central Sierra Nevada had the greatest increase in LFD (Figure 10). Changes in MAF were largely driven by area and increased evapotranspiration from climate warming, CT shifts were attributable to snowfall and snowmelt timing, and LFD was driven by deep soil moisture capacity and infiltration. (Climate warming impacts in Figure 10 are values of reduced MAF per square kilometer, or change in number of weeks for CT and LFD, scaled by quartiles for comparison.)

A few watersheds had large changes from more than one metric (MAF, CT, or LFD), and are thus more vulnerable to climate warming than surrounding watersheds. For example, the American and Mokelumne watersheds are almost always in the 4^th^ quartile (the most change from basecase conditions). The Kern watershed is notable because model results indicate that it is consistently more resilient to climate warming. Its high elevation protects it from more of the effects of climate warming than other watersheds in the range.

### Climate Warming Impacts on Water Resources

Water yield and timing changes in each watershed imply that water resource developments and operations will be affected with climate warming [26]. For instance, climate warming is expected to raise the snowline elevation and increase the likelihood of warm storms, such as pineapple express storms, with more rainfall runoff (rather than spring snowmelt) [27], and more rapid spring snowmelt at upper elevations [28]. While this study modeled changes to runoff from climate warming, it did not include infrastructure (i.e., storage reservoirs), and therefore we did not examine changes in storage or uncontrolled releases from reservoirs. It was assumed that the water discussed here could be captured and delivered to existing water users. Thus, the results discussed here can be interpreted as an upper bound of water yield for water supply, hydropower, recreation, and environmental protection. It is probable that if infrastructure were included, model results would indicate even larger reductions in MAF because more water would be lost as uncontrolled storm flows from flashier storms as well as greater evaporation from reservoirs. Research on climate change related flow reduction for study watersheds with regulated conditions exists for some watersheds [9], [26], but is hard to compare because different locations, time periods, or climate change scenarios were modeled.

To measure intrinsic vulnerability across the study system – and to elucidate broad trends that focus climate warming adaptation strategies – we compare unimpaired change in MAF to total water storage, unimpaired change in CT to total hydropower capacity, and unimpaired change in LFD to mountain meadow area. For this paper, we define intrinsic vulnerability of a watershed as the inherent ability of the system to cope with external, natural, and anthropogenic impacts that affect its state and character in space and time (adapted from [29]).

#### Water Storage

**Figure 11:**
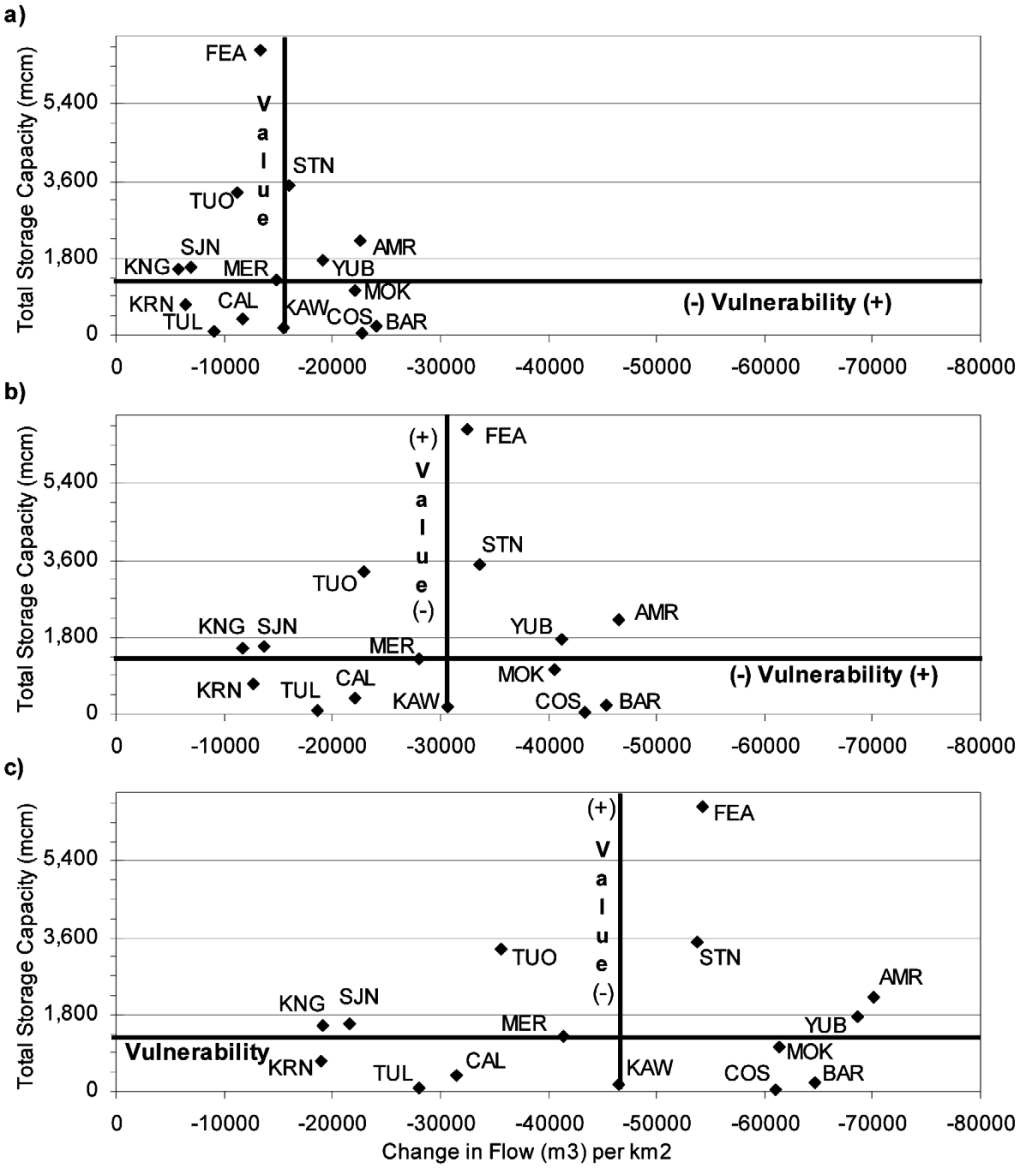
Relative vulnerability of watersheds based on total water storage and change in MAF for a) 2°C climate warming, b) 4°C climate warming, and c) 6°C climate warming.

Changes to MAF within each watershed impact water supplies for downstream urban, agricultural, and environmental water supplies. Unimpaired MAF change per square kilometer was compared to total water storage within each basin for 2°C, 4°C, and 6°C warming (Figure 11). Value and vulnerability axes were placed on the median values for all the watersheds, so that half the remaining watersheds had more water storage capacity and reduction in MAF. The watersheds in the top portion of each graph are those with the most water storage, and thus were assumed to have most value to society. The watersheds on the right side of each graph are those that had the greatest reduction in MAF, so were assumed to be the most vulnerable to climate warming. The watersheds in the upper right quadrant (resulting in the bisection of the two medians) are those that are both valuable for water storage and most vulnerable to climate warming.

Overall, few watersheds changed quadrants with increased climate warming in Figure 11; although climate warming reduced MAF for all watersheds. The American watershed had the greatest change in MAF for 4°C and 6°C warming, although the Bear and Cosumnes watersheds had greater reductions in MAF for 2°C warming. These watersheds, along with the Yuba and Mokelumne Rivers always had the largest reduction in MAF km^−2^, regardless of the extent of climate warming. Of those five watersheds, the American and Yuba River watersheds are fairly valuable for water storage, and the Mokelumne, Bear, and Cosumnes watersheds have relatively little total water storage. Most rivers did not change quadrants, and kept their position relative to other rivers. The Kings and Kern Rivers always had the least reduction in MAF. The Feather, and to a lesser extent the Stanislaus and Tuolumne watersheds, all have significant water storage capacity, and remained near the median for vulnerability to climate warming.

Ignoring water supplies, the watersheds on the right side of Figure 11 are those that model results suggest will have the largest reduction in flow volume from climate warming, which also affects instream conditions and habitat for aquatic and riparian ecosystems. Thus, the watersheds on the right side of Figure 11 could be expected to have more environmental change as well. This implies the American, Yuba, Bear, Mokelumne, and Cosumnes Rivers may have the most altered aquatic and riparian ecosystems under all climate alternatives. These watersheds are all in the northern Sierra Nevada, indicating this sub-region may have greater flow reductions from climate warming than surrounding watersheds, which would likely stress traditional water uses for irrigation and urban water storage, and as well as aquatic and riparian ecosystems. Additional habitat losses are likely for native aquatic species in the northern extent of the Sierra Nevada.

#### Hydropower Generation

**Figure 12:**
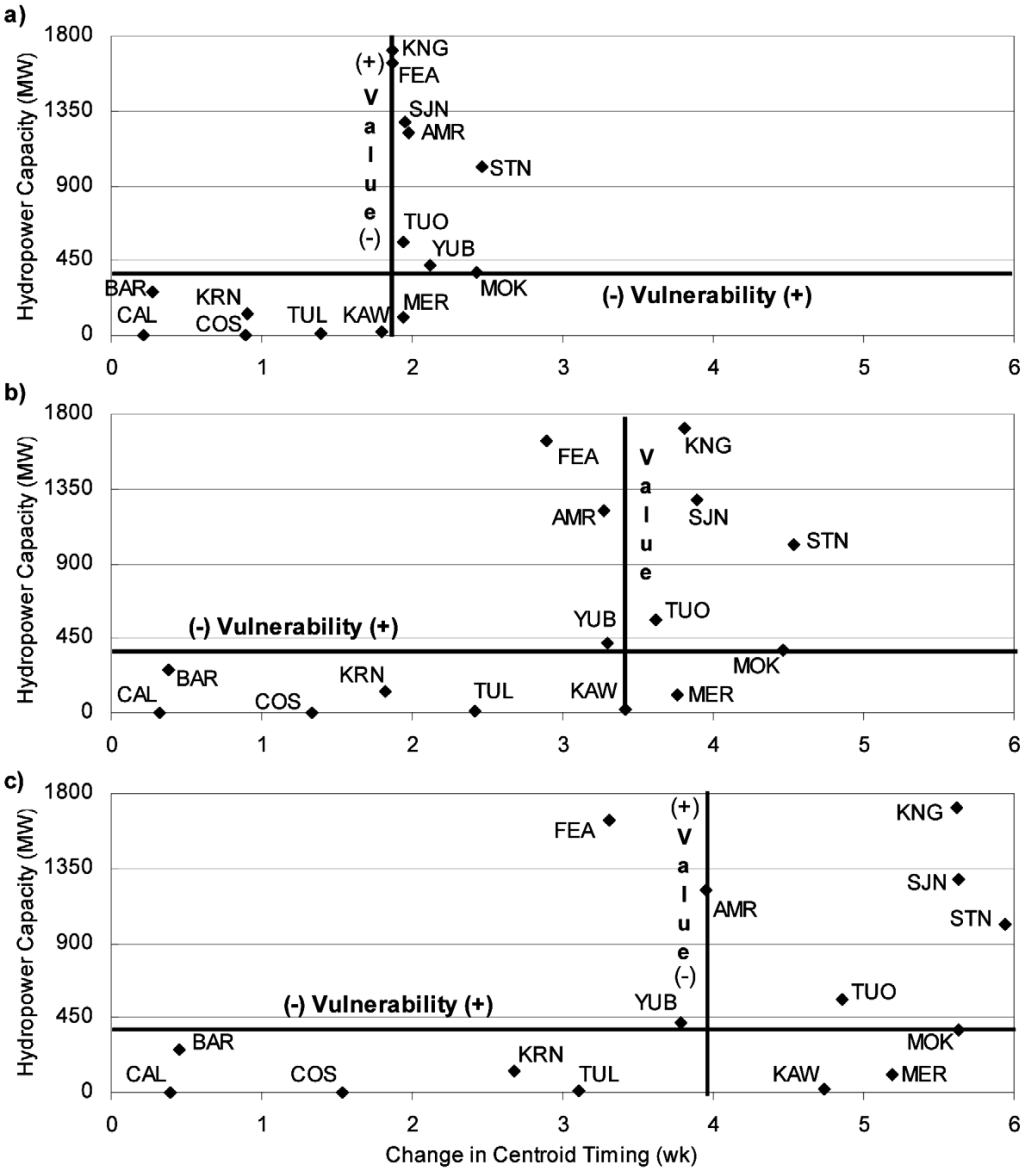
Relative vulnerability of watersheds based on total available hydropower capacity and change in CT for a) 2°C climate warming, b) 4°C climate warming, and c) 6°C climate warming.

Changes to seasonal runoff timing were compared with hydropower capacity for each basin (Figure 12), although timing changes from climate warming may also affect flood protection, water storage, and deliveries. Online hydropower capacity was used here, which is the maximum generating capacity for each facility. Watersheds that were both valuable and vulnerable are watersheds that we rely on for hydropower generation and that may face substantial changes in runoff timing with climate warming. Hydropower is often generated during high demand periods (e.g., seasonal summer peaking operations), which may be compromised if facilities are forced to spill due to higher magnitude flows or to accommodate earlier arrival of flows. Total current hydropower online capacity is on the y axis of all plots in Figure 12 and does not change with climate warming. Value and vulnerability axes were placed on the median watersheds for CT and hydropower capacity, respectively. Similar to the MAF vulnerability figure, watersheds in the upper, right quadrant were both valuable for hydropower generation and vulnerable to runoff timing changes associated with climate warming.

Figure 12 is notable because watersheds with the most hydropower capacity were also those with the greatest shift in CT with climate warming, and those with the lowest capacity for hydropower production were the least vulnerable to CT change. As such, most watersheds are located either in the upper right quadrant or lower left quadrant. The Kings, San Joaquin, Stanislaus, and Tuolumne all have capacity to produce considerable hydropower and were consistently vulnerable to runoff timing change.

Watersheds changed position from changes to CT more than from changes to MAF. The Stanislaus and Mokelumne were always in the top three watersheds with the most change to runoff timing. The Tule, Kern, Cosumnes, Bear, and Calaveras consistently had the least change in seasonal runoff. All other watersheds changed their ranking for CT with various degrees of climate warming.

#### Mountain Meadows

**Figure 13:**
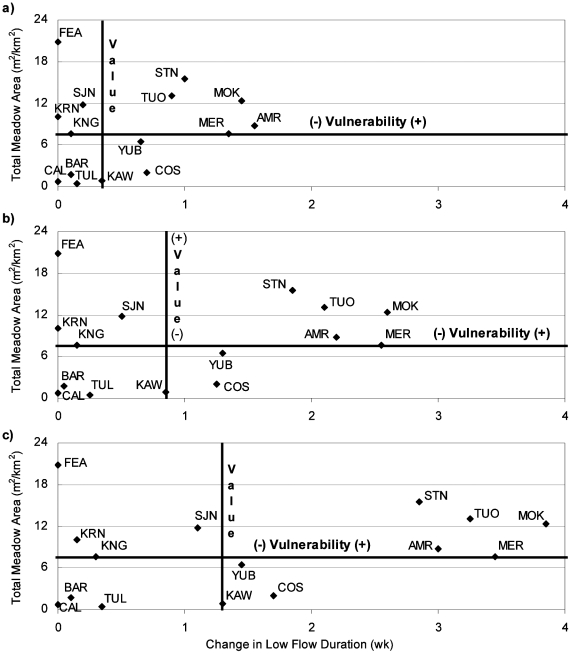
Relative vulnerability of watersheds based on meadow area per square kilometer and change in LFD for a) 2°C climate warming, b) 4°C climate warming, and c) 6°C climate warming.

We compared LFD in each watershed with mountain meadow area (m^2^/km^2^) (Figure 13), which is used here as a surrogate for montane ecosystems. We assumed that persistent low flow conditions deplete meadow groundwater reserves and soil moisture, reducing the downstream benefits of meadows. Meadows provide many ecosystem services such as maintaining summer flow during dry periods and reducing floods in winter [30]; providing aquatic and riparian habitat for birds, fish, amphibians, and bugs [31]; promoting riparian vegetation rather than conifer or dry shrub vegetation that increase wildfire risk [32]; and improving downstream water quality [33]. Mountain meadow health and abundance is one of many ecosystem services that could be degraded with future climate warming.

Figure 13 shows low flow duration on the horizontal axis, and meadow area normalized by watershed area on the y-axis. Vulnerability and value markers were placed on the median watersheds. Like previous figures, the watersheds in the upper, right quadrant are those that are both valuable for the ecosystem services provided by meadows, and vulnerable to lengthened LFD.

Overall, watersheds did not change quadrants in the LFD vulnerability figure with increasing climate warming (Figure 13). The American River was most vulnerable to increased LFD with 2°C warming, although became less vulnerable relative to other watersheds (Mokelumne, Merced, and Tuolumne) with a warmer climate. The above watersheds, along with the Tuolumne watershed, remained in the upper, right quadrant, indicating mountain meadows and associated ecosystem services may decline in those basins. Likewise, the Calaveras, Bear, and Tule Rivers remained in the lower, left quadrant, because they have relatively little meadow area and modeling suggests they were more resilient to LFD. In fact, the Calaveras, Bear, Tule, Cosumnes, and Kaweah are all watersheds that do not extend to the crest of the Sierra Nevada, and had the least amount of mountain meadow area.

## Discussion

Although it has been well documented that climate change is likely to increase air temperature and reduce snowpack in California's Sierra Nevada [1], [5], [28], few studies have examined the differential impacts of climate warming for neighboring watersheds. This is a major information gap, leading to a general absence of climate change planning at the local to regional scales within an intrabasin comparative framework. Water resource managers will be impacted by future climate warming, and may have to anticipate climatic changes as a component of resource management. Overall, few water agencies have released planning documents that address climate warming in a specific manner, and that include discussion of potential operational changes. At present, climate change impacts are also not considered in the FERC relicensing process, although water projects will probably affect aquatic ecosystems and other river resources differently with a warming climate. Water for hydropower generation may runoff earlier in the year, although power demand will likely increase in California with a warmer climate. In densely populated regions as well as those with extensive farmland, water utilities must adapt to coming climate changes to provide reliable water supplies.

This paper responds to scientific uncertainty by modeling climate warming impacts on the watershed scale to allow water resource managers to understand general trends and appropriately guide their adaptation strategies. Results suggest that watershed response to climate warming is not homogenous throughout the Sierra Nevada. Overall, watersheds in the northern Sierra Nevada are more susceptible to reductions in MAF, the high elevation watersheds in the southern-central region are most susceptible to earlier runoff timing, and those in the central Sierra Nevada are most vulnerable to longer low flow periods. Modeling indicates that the American and Mokelumne watersheds are among the most vulnerable to all three of the MAF, CT, and LFD metrics, and the Kern watershed is the most resilient. In WEAP, MAF changes were driven primarily by ET and area, CT was driven by snowmelt volume and timing, and LFD was driven by soil moisture, particularly in the deep soil layer.

Additionally, some of the most valuable watersheds for water resources and ecosystem services are those that are most affected by climate warming. The American, Yuba, and Feather watersheds are developed extensively for water storage, although these basins were predicted to experience considerable reductions in flow. The Stanislaus, Kings, and San Joaquin all have substantial hydropower capacity, and results suggest spring runoff may occur approximately 6 weeks earlier with 6°C climate warming. A significant portion of the Mokelumne, Tuolumne, and Stanislaus watersheds are mountain meadows, although these watersheds also had increases in the length of low flow conditions with climate warming. Finally, the estimates included in this paper should be considered an upper bound (or best case scenario) because uncontrolled losses and evaporation from reservoirs were assumed to be zero. Hydrologic changes from climate warming are also expected to impact aquatic ecosystems, habitat availability, and ecosystem services not incorporated here. A warming climate will likely further stress aquatic ecosystems, which have already undergone extensive habitat loss from the water resource development and land use changes in the Sierra Nevada. Downstream flood protection was also not considered for this research, although rising snowline elevations are expected to increase the magnitude and frequency of storm events, often increasing the probability of catastrophic flooding, similar to the 1997 water year.

Incorporating the changes and uncertainties associated with climate warming into water resource management and policy will not be easy. The Federal Energy Regulatory Commission (FERC) regulates non-federally owned hydropower projects, providing one of the only formal opportunities to reduce and mitigate impacts to other non-power water users (i.e. water supply, environmental protection, recreation) through license conditions and settlement agreements. However, FERC currently does not consider climate change in the licensing process, despite FERC licenses lasting 30–50 years [34]. Incorporating climate change into the FERC licensing process provides one policy opening to highlight hydrologic uncertainty and changing trends for protection of hydropower, water supply, and environmental benefits, and to avoid narrow, inflexible operations that will not be compatible with altered and more variable hydrologic conditions.

California's water resources have been extensively developed and for this reason are routinely studied in their own right [3], [4], [5], [6], [9], [10]. However, many of the findings are applicable to other mountain regions. Climate warming is expected to have severe impacts on mountain regions throughout the world [35]. In coming decades, as climate warming affects existing water resource management in mountain regions, attention will focus on how to adapt resource use to maintain traditional water uses, while providing adequate flood protection, and ensuring aquatic and riparian habitat for dependent ecosystems. This study helps to shed light on the types of changes that mountain regions will face, the drivers of change within basins, the variability between neighboring watersheds, and potential effects to highly populated downstream areas.
